# Tenofovir-associated kidney disease in Africans: a systematic review

**DOI:** 10.1186/s12981-019-0227-1

**Published:** 2019-06-06

**Authors:** Takudzwa J. Mtisi, Chiratidzo E. Ndhlovu, Chiedza C. Maponga, Gene D. Morse

**Affiliations:** 1 Department of Clinical Pharmacology, University of Zimbabwe College of Health Sciences, Harare, Zimbabwe; 2 Department of Medicine, University of Zimbabwe College of Health Sciences, Harare, Zimbabwe; 3 School of Pharmacy, University of Zimbabwe College of Health Sciences, Harare, Zimbabwe; 4 Center for Integrated Global Biomedical Sciences; School of Pharmacy and Pharmaceutical Sciences, University at Buffalo, State University of New York, Buffalo, NY USA

**Keywords:** Tenofovir, Renal, Africans

## Abstract

**Background:**

Data on chronic kidney disease development in HIV infection is important towards building a comprehensive knowledge of HIV, ageing and polypharmacy in Africa. Several previous studies on tenofovir-associated kidney disease in Africa have shown conflicting results. This review summarises what is known about the development of kidney disease in HIV-positive African patients on tenofovir disoproxil fumarate (TDF)-containing ART. We set out to document the occurrence of kidney disease in HIV-positive Africans on TDF-containing ART in population-based studies and to evaluate the renal safety of TDF in Africans.

**Methods:**

We conducted a systemic review using published studies which were identified through a computerized search of original research using the Medline/PubMed database, EMBASE, EBM Reviews, Proquest Google Scholar and Global Health reported from inception until 5 October 2017. Two reviewers independently abstracted the data and performed quality assessment of the included studies. We screened 595 articles and included 31 in the qualitative analysis performed.

**Results:**

A total of 106 406 patients (of whom 66,681 were on Tenofovir) were involved in these 31 studies with sample sizes ranging from 30 to 62,230. Duration on tenofovir-containing ART ranged from those initiating ART at baseline to those who received TDF for up to 9 years. All but one of the studies involved only patients 16 years and older. The studies had differing definitions of kidney dysfunction and were of variable study design quality. The documented outcomes had substantial discrepancies across the studies, most likely due to methodological differences, study size and disparate outcome definitions.

**Conclusions:**

Our review identified studies in Africans reporting statistically significant renal function decline associated with TDF use but the clinical significance of this effect was not enough to contraindicate its continued use in ART regimens. Consistent with studies in other populations, patients are at greater risk if they have pre-existing renal disease and are more advanced in age. More research is needed on paediatric populations under 16 years of age.

*Trial registration* This review was registered on Prospero (registration number CRD42018078717).

**Electronic supplementary material:**

The online version of this article (10.1186/s12981-019-0227-1) contains supplementary material, which is available to authorized users.

## Background

Tenofovir disoproxil fumarate (TDF or commonly termed “tenofovir”) is a Nucleoside Reverse Transcriptase Inhibitor (NRTI) that acts by blocking reverse transcriptase, an enzyme critical for viral production in HIV-infected people. It is still widely used as part of first-line antiretroviral therapy (ART) in resource limited settings such as in Africa. It is administered orally and is widely distributed with the highest concentrations occurring in the kidney and liver [1, 2]. The major route of elimination is through the kidneys via glomerular filtration and tubular secretion and its clearance is in the proximal tubule of the nephron is controlled by active transport [1]. Higher tenofovir plasma levels result in intracellular accumulation in the renal tubular cells a consequent increased risk of renal toxicity [1]. Genetic variation in these transporters may also influence exposure of the kidney to tenofovir, hence play a role in tenofovir associated renal toxicity [2–7].

While TDF has been shown to be effective and relatively safe, several studies indicate that it has nephrotoxic potential, characterised by proximal tubular cell injury which may result in acute kidney injury (AKI), chronic kidney disease (CKD) or partial or complete Fanconi syndrome [5, 8–11]. This may compound HIV associated nephropathy (HIVAN), a condition which is a leading cause of chronic kidney disease and end-stage renal disease (ESRD) and is caused by direct injury to the kidneys by the Human Immunodeficiency Virus (HIV) [12]. HIVAN is documented as being more common in African Americans that their white counterparts but has wide variability in different Sub-Saharan populations [13, 14]. AKI usually results in discontinuation of the drug while chronic manifestations may be managed by closer monitoring of the patient and treating symptomatically. Regardless of the underlying aetiology of kidney disease, if left untreated, it may lead to death [15]. Initiating patients with reduced estimated glomerular filtration rates (eGFR) of < 50 ml/min/1.73 m^2^ on TDF containing ART has been shown to increase the risk for renal dysfunction in the said patients [15]. In developed countries, although ART continues to be given to patients with renal disease, if indicated, most NRTIs must be dosed according to renal function and some ARVs are avoided [14, 16].

Several studies conducted in Africa provide conflicting evidence on the renal outcomes of HIV-positive African patients on TDF. This necessitated a review that provides a resource to summarise specific data required for objective decision making on the renal safety of TDF in African populations.

### Rationale

The advent of anti-retroviral therapy has resulted in patients living longer with HIV. However, this is not without consequence. As these patients age, they are at increased risk of developing chronic conditions such as hyperlipidemia, cardiovascular disease (CVD) and chronic kidney disease (CKD). The consequent polypharmacy in trying to manage the multiple conditions in the patient, which includes use of medicines like TDF reported to cause renal disease may also further compound the burden on the HIV positive patient. Debate continues over whether widespread use of TDF, particularly in “real world” clinical settings, poses a risk for nephrotoxicity significant enough to limit its use, to necessitate close clinical monitoring or to identify high risk patients at initiation and closely monitor them [11, 17, 18]. In recent years, the option of tenofovir alafenamide (TAF) has arisen as it is documented to have a safer renal profile than TDF. TAF has a similar tolerability, safety, and effectiveness to TDF and probably less adverse events related to renal and bone density outcomes in the treatment of naive and experienced patients with HIV-1 [19]. Given that TAF is documented as having higher viral suppression rates and better renal safety and bone density safety profiles, it has better clinical advantages over TDF and could be considered to replace TDF. Knowing the extent of renal safety of TDF in low resource settings (LRSs) would inform policy as to the need or priority to change patients to TAF.

The data on co-morbidity in HIV infection is especially important when considering HIV in Africa coupled with ageing and polypharmacy and the implications in such resource limited settings. We reviewed the existing literature on acute kidney injury and TDF-associated nephrotoxicity not to provide a systematic literature review with weighted evidence, but rather to provide a collated source of evidence from available sources. We aimed to summarise what is known about the development of kidney disease in HIV positive African patients on TDF containing ART, from verified data sources. The specific objectives were:

- To document the occurrence and patterns thereof, of kidney disease in HIV-positive Africans on TDF-containing ART in population-based studies.
- To evaluate the renal safety of TDF in African HIV positive patients.

## Methods

### Study design

A systematic review was conducted according to the Preferred Reporting Items for Systematic Reviews and Meta-Analyses (PRISMA) group [20].

### Eligibility criteria, data sources and study selection

We performed a systematic search of English articles from the following electronic databases from inception to the date of search as indicated: Pubmed (5 October 2017), Embase (5 October 2017), EBM reviews (5 October 2017). We also considered grey literature in the form of reports of original studies, unpublished master’s thesis and PhD dissertations written in English in ProQuest Dissertations and Thesis Global database. Meeting abstracts archives of International AIDS Conference (IAS), International Conference on AIDS and Sexually Transmitted Infections in Africa (ICASA), African Society for Laboratory Medicine (ASLM) were also searched for any relevant unpublished work as long as they reported renal outcomes of African HIV patients on TDF. We included data from primary research of cross-sectional studies, observational cohort, case–control studies and randomized control trials reporting renal outcomes of HIV positive patients on a TDF-containing regimen. Studies done on Africans residing on the continent satisfying the PICOS criteria in Table 1 were included. Articles that were not written in English, commentary, editorials, reviews, publications in duplicate and articles only available in abstract form were not included in the review. Selection of articles was done in three phases: titles alone, abstracts, and then full text articles.

**Table 1:** Inclusion criteria

Study design	Cohort
	Case–control
	Cross-sectional (if duration of TDF therapy is stated)
	RCT
Article characteristics	Full articles
	Open access dissertations/theses
	Grey literature
Participants	Africans
	All age groups
	HIV positive on TDF containing ART
Setting	Primary research done on the African continent
Intervention	TDF containing ART
Outcome	Kidney dysfunction. Indicated by any of the following outcome measurements:
	Glycosuria, phosphaturia
	CrCl and eGFR data
	Serum creatinine data
	Albumin creatinine ratio
	Protein creatinine ratio

### Search

The following search terms were used: (“Kidney dysfunction” OR “Kidney impairment” OR “Kidney failure” OR “Renal disease” OR “Renal dysfunction” OR “Renal impairment” OR “Renal failure”) AND (“HIV seropositive”) AND (TDF OR “Tenofovir Disoproxil Fumarate”) AND (“Antiretroviral therapy” OR “ART” OR “Highly Active Antiretroviral therapy”) AND (Africa OR Africans OR Blacks OR “Black Africans”). The terms were adjusted as appropriate for searching in each respective database (Additional file 1).

### Data abstraction

Two reviewers (TM and AH) independently reviewed all titles and abstracts of the search results in two phases. The retrieved titles and abstracts were reviewed first to identify relevant studies against the inclusion criteria in Table 1.

The study eligibility criteria checklist was piloted on ten publications to check for consensus interpretation and classification of studies. Full texts of selected studies were then retrieved and read to determine eligibility for inclusion in the qualitative analysis. A PRISMA study flow diagram of included and excluded studies was developed showing reasons for exclusion (Fig. 2).

A data extraction form designed to focus on population, study design, methodology and outcome was developed. One reviewer used this to extract the data from all the studies identified at screening (TM). A second reviewer (AH) checked for errors. Three discrepancies were noted, discussed, resolved by consensus and amended as required. Microsoft Excel was used for the management of data.

### Risk of bias assessment

We used the NIH Quality Assessment Tool for Cohort and Cross Sectional Studies (National Institutes of Health, 2014) checklist. Each of the 16 items on the checklist was assigned a score of 1 (yes) or 0 (no or CD, cannot determine; NA, not applicable; NR, not reported). Scores were then collated across items to give an overall quality score ranging from 0 to 16. Each of the studies was then rated as being of low, moderate, or high methodological quality depending on the number of questions answered as “yes (low risk of bias)”. Studies were deemed high quality if they had scores higher than 13, moderate if they had a score of 8–12, and low a score of 7 or lower.

### Ethics statement

Since this research was a systemic review based on the data extraction of published articles, ethical approval was not necessary and was therefore, not sought.

## Results

### Identified studies (Fig. 1)

**Fig. 1:**
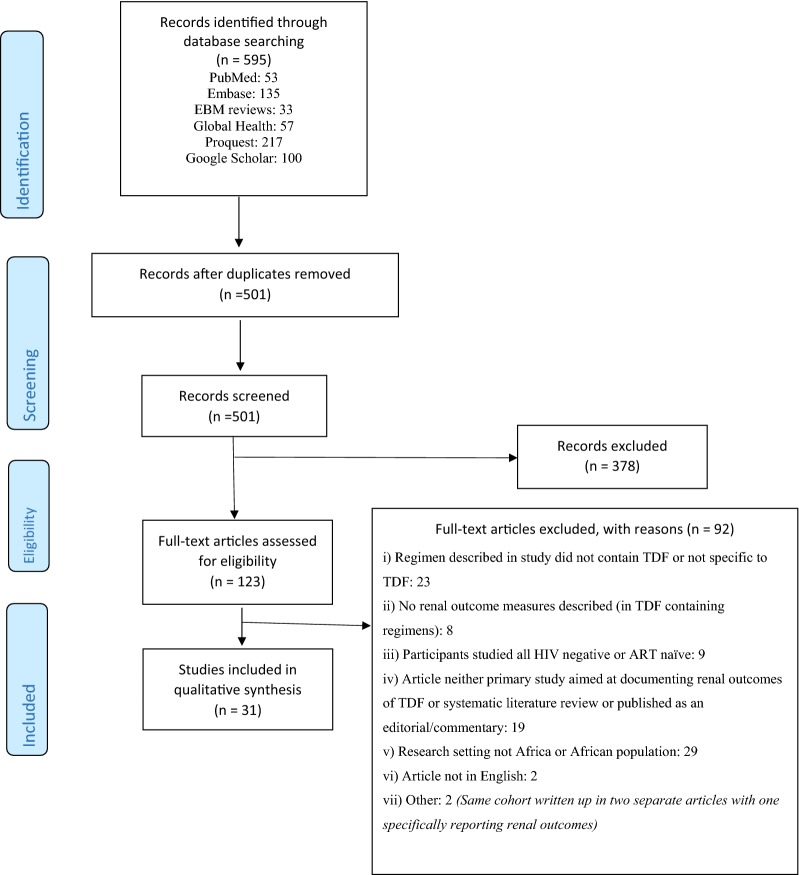
Literature search results

We retrieved a total of 595 articles from six databases namely Pubmed Central, EMBASE, EBM Reviews, Global Health, Proquest and Google Scholar. Ninety-four duplicates were removed after merging the articles. Of the remaining 501 articles, 378 were removed on the basis of ineligibility of their titles and/or abstracts to satisfy the inclusion criteria as defined in Table 1. One abstract fit the inclusion criteria but no manuscript was available for analysis and hence the abstract was excluded [21]. Full text articles were then retrieved for 123 articles of which 92 were excluded for reasons specified in Fig. 1 leaving 31 suitable articles for this review that included a total of 106 406 participants.

### Characteristics of included studies (Table 2)

Of the 31 studies included in this analysis, 18 were cohort studies, 11 of which were retrospective cohorts [15, 22–39]. Seven studies were cross-sectional [40–46], two randomized control trial (RCT) [47, 48], two observational analyses within RCTs [49, 50], one prospective case control study [51] and one was in the form of targeted spontaneous reporting within the context of implementation research [52]. Sample sizes ranged from 30 to 62 230. The duration on tenofovir-containing ART ranged from 0 to 9 years.

**Table 2:** Characteristics of included studies

Author: year	Setting/Region*	Age group of interest	Design	Sample size (on TDF containing regimen)	Treatment regimen	Comparator	Duration on ART at baseline	Duration of follow up	Outcome measurements
Mulenga: 2014	Zambia: SA	≥16 years	RC	62,230 (38,716)	TDF-based regimen	Non TDF containing	–	12 months	eGFR by CKD-EPI
De Waal: 2017	South Africa: SA	≥ 16 years	RC	15,156 (15,156)	TDF containing	–	Median 12.9 months	–	eGFR using MDRD, CKD-EPI and CG. No factor for black race used
Ndagije: 2015	Uganda; EA	Adults	Implementation: (TSR)	10,225 (53)	TDF based	–	> 2 years	2 years	SCr, urinalysis, clinical signs and symptoms
Reid: 2008	DART: Uganda and Zimbabwe	≥16 years	OC within RCT	3316 (2469)	TDF/3TC/AZT	Non TDF	Naïve at baseline	96 weeks	Creatinine levels and eGFR by CG
Stöhr: 2011	South Africa: SA	Adults	OC within RCT	3316 (2469)	TDF/3TC/AZT	Non TDF	Naïve at baseline	96 weeks	eGFR by CG
Shamu: 2015	Zimbabwe: SA	Adults ≥ 18yrs	RC	1986 (1862)	TDF containing	Non TDF		6411 person yrs	Two consecutive CrCl < 60 ml/min by the CG equation, or two consecutive urine dipsticks positive for protein (≥ 30 g/L albumin)
Myer, 2013	South Africa: SA	Women ≥ 25 years	RC	1861 (238)	TDF, 3TC/FTC, and EFV	Men and non-pregnant women, similar regimen	initiating Rx	2 years	CrCl and absolute serum creatinine values
Dekert: 2017	Zambia; SA	≥16 years	RC	1118 (1118)	TDF containing	–	≥ 90 days	Median 1461 days	eGFR, CKD-Epi formula
Mayanja: 2017	Uganda; EA	Adults	PC	1095 (608)	TDF containing	236 on a PI + other	≥6 months, Median 9.4 years		sUrea, sCr, Fractional Tubular phosphates reabsorption & eGFR. (CG/MDRD/CKD-Epi)
Kamkuemah: 2015	South Africa: SA	Adults	Cohort	1092 (1092)	TenolamE	–	initiating ART	12 months	CrCl after 12 months on ART measured by glomerular filtration rate (eGFR) using CG
Salome: 2016	Uganda; EA	Adults	CS	953 (568)	TDF containing	385 non TDF	≥ 6 months. Median 9.3 years	Cross sectional	eGFR by CKI-Epi
Bygrave: 2011	Lesotho: SA	Adults	RC	933 (566)	TDF containing	–	pts initiating TDF ART	12 months	CrCl using CG
Brennan: 2011	South Africa: SA	Adults	RC	890 (890)	TDF-containing	–	pts initiating TDF ART	48 months	Nephrotoxicity defined as decline in kidney function from baseline secondary to a toxin. CrCl to estimate eGFR using CG
Mwafongo: 2014	Eastern and Southern Africa:	Women ≥ 13 years	RCT	741 (741)	TDF containing	–	TDF naïve at baseline	48 weeks median 2.3 years	CrCl or clinical renal diagnosis
Zachor : 2016	South Africa: SA	Adults	RC	650 (650)	TDF containing	–	ART naïve	median 47 w	eGFR by CKD-EPI and confirmed by MDRD
Mugomeri: 2014	Lesotho: SA	Adults	RC	485 (312)	TDF containing	173 on other	> 6 months	> 6 months	eGFR by CG
Zannou: 2015	Benin; WA	Adults ≥ 16yrs	CS	480 (62)		2 NRTIs + 1 NNRTI. Those on 2nd line comprised 2 nucleosides RTIs + 1 PI	> 3 nths	Cross sectional	CrCl by CG
Wantakisha: 2017	Zambia: SA	Adults ≥ 15 years	CS	445 (445)	TDF containing	−	18 months	Cross sectional	CrCl
Cournil: 2016	Cameroon, Burkina Faso, senegal	Adults	RCT	438 (306)	A: TDF + FTC + LPV/r (152); C: TDF + FTC + darunavir (DRV)/r (154)	B: ABC + ddI + LPV/r	18 months on 2nd line	18 months	eGFR (MDRD)
De Beaudrap: 2010	Senegal: WA	Adults	PC	428 (40)	TDF-containing	Non-TDF	pts initiating on ART	42 months	eGFR using CG and MDRD
Chadwick: 2015	Ghana: WA	Adults	CSO	330 (101)	TDF containing	Either AZT or D4T + NVP or EFV	≥ 6 months, 20 months median time for TDF	Cross sectional	CrCl using CG, dipstick proteinuria, uPCR, uACR, uAPR, fractional phosphate and urate excretion. TD defined as having ≥ 2 of: fractional phosphate excretion > 18%, fractional urate excretion > 15%, normo-glycaemic glycosuria, proteinuria (uPCR > 20 mg/mmol) with uAPR < 0.4
Banda: 2010	Zambia; adults	Adults	CS	300 (52)	TDF containing	D4T (3), AZT (6), HIV- (158)	Not stated	Cross sectional	RD defined as rise of sCr to 1.5 × upper limit of normal i.e. ≥ 180 umol/L
Orluwene: 2015	Nigeria: WA	≥ 16 years	PCC	254 (100)	100 TDF based	102 Non-TDF. 52 Rx naïve	…	…	Urinary interleukin (Il)-18, eGFR by CG/MDRD/CKI-Epi
Mpondo: 2014	Tanzania; EA	Adults	RC	238 (54)	54 TDF	184	–	Median 2 yrs	eGFR by CG/MDRD
Agbaji: 2011	Nigeria; WA	Adults	OC	186 (84)	TDF containing	Non TDF	≥ 48 weeks	> 48 weeks	sCr and CrCl using CG
Fritzsche: 2017	Cameroon; WA	Adults	CS	179 (119)	TDF containing	ART naïve pts	Mean period of 301 ± 193 days	Cross sectional	Urinary dipstick for proteinuria, sCr, eGFR using CKD-EPI. Kidney size and structure by ultrasound, renal echogenecity grading as described by Garko
Seedat: 2017	South Africa: SA	≥ 15 years	PC	175 (93)	93 TDF exposed,	TDF unexposed	…	Duration of hospitalisation.	AKI by (KDIGO) 2012 Clinical Practice AKI Guideline. sCr eGFR by MDRD
Kalemeera: 2016	Namibia: SA	Adults	RC	71 (71)	Second line TDF containing ART.	–	Mean 5.2 years on 1st line, 1.8 years on 2nd line	Cross sectional	CrCl. Renal function categories based on CG
Gajee: 2016	South Africa: SA	Adults 20–40	RC partial prospective	66 (66)	TDF containing	–	12 months	12 months	eGFR by CG at before TDF commencement and 12 months post-TDF commencement.
Tewogbade: 2010	Nigeria;WA	Adults	PC	55 (19)	TDF + 3TC + EFV. or as truvada	D4T or AZT	initiating ART	12 months	CrCl (MDRD), eGFR by CG
Mulubwa: 2016	South Africa: SA	Adult women	CS	30 (30)	300 mg TDF nocte	30 HIV uninfected	–	Cross sectional	eGFR (MDRD), CrCl by CG

Designs: *OC* observational cohort, *RC* retrospective cohort, *PC* prospective cohort, *CS* cross sectional, *RCT* randomized control trial, *TSR* targeted spontaneous reporting, *PCC* prospective Case Control Regions of Africa: *CA* Central Africa, *EA* Eastern Africa, *SA* Southern Africa, *WA* West Africa

While TDF was included in the ART regimens in all the studies, there was wide variation in ART combinations and concurrent medications and durations thereof. Not all studies reported concurrent medications. Ten studies [15, 23, 24, 29, 32, 35, 39, 47, 49, 50] recruited only participants who were TDF naïve at baseline while the rest recruited patients who were TDF experienced for at least 6 months. The median age of the participants across all the studies ranged from 34 to 43 years with all studies including more females than males. All studies except one included only adult patients with the one that included patients aged **≥** 13 years being pregnant females. Less than 50% of the studies reported CD4 counts at the baseline, at study end or both. Of those that did, baseline CD4 counts were low (< 200) at baseline and for those that reported at study end, these had improved.

### Renal parameters reported

Table 2 illustrates that renal outcomes were variably defined across the studies. In about 60% of the included studies, eGFR (using one of or a combination of CockCroft-Gault, MDRD, CKI-EPI formulae) was used as a measure of renal outcomes. Another 33% reported at least Creatinine clearance (CrCl) as the outcome measurement [22, 25, 28, 30–32, 49–51]. Only two studies by Zachor et al. and Banda et al. [40, 52] reported only serum creatinine (sCr) with Zachor defining renal dysfunction as the rise of sCr to 1.5 times the upper limit of normal, i.e. ≥ 180 umol/L and Banda reporting sCr, urinalysis, clinical signs and symptoms as measures of renal dysfunction. In addition to reporting CrCl, Myer et al. also reported absolute serum creatinine values [36].

### Renal safety outcomes

Table 3 summarises the findings and conclusions made by the various authors from their studies. About 90% of the studies focused on chronic outcomes of kidney function in patients on TDF-containing ART while two reported on acute outcomes [34, 35, 51]. Most of the studies reported at least some incidence of RD in the patients taking TDF containing ART. Fifteen report overall safety of TDF in ART regimens, recommending its continued use with monitoring [28–31, 37–40, 42, 44, 46, 48–50]. Although the definitions of renal dysfunction differed widely, the other 16 studies report either statistical or clinical association of TDF with renal dysfunction [15, 22–26, 32–35, 43, 45, 47, 51–53]. Groups at higher risk include patients with impaired renal function at baseline, older age groups and women [33, 35, 47]. Mulenga et al. and Mwafongo et al. suggest that development of renal dysfunction is more likely during the first year of using TDF. Zachor et al. report that the risk of developing stage 3 CKD increases by up to 1.9-fold for every 10 year increase in age and women are four times more likely to develop end stage CKD than their male counterparts. Further, three studies focused on pregnant women; all reporting different outcomes from each other.

**Table 3:** Summary of findings for each study

Author: year	Findings	TDF relationship conclusion by author	Effect size/clinical significance
Banda: 2010	TDF was not associated with RD (1.03: 0.45–2.37, 95% CI)	TDF not associated with RD	N/A
Fritzsche: 2017	Protenuria was significantly more prevalent, and creatinine was significantly higher among treatment naive than among those on treatment (52.2% vs 26.1%; p = 0.003 and p = 0.009 respectively. The proportion of pts with an eGFR < 60 ml/min was significantly higher among treatment naive pts than among those on TDF treatment (40.4% vs 24.4%; p = 0.041). Treatment naive pts displayed an improvement in Cr levels and eGFR after 6 months of treatment	TDF appears to be safe and does not appear to be a significant cause of renal impairment	N/A
Kalemeera: 2016	There was no difference between TDF based and non TDF 1st line ART, on the CrCl (95% CI: 102[94–111] vs 95[87–102] ml/min; p = 0.78. In addition, the type of 1st line whether TDF containing or TDF free did not influence the CrCl during second line ART (95% CI 105[96–114] vs 96[87–104] ml/min; p = 0.90	No difference between TDF based and non TDF 1st line ART, on the CrCl. In addition, the type of 1st line whether TDF containing or TDF free did not influence the CrCl during second line ART. The presence of renal impairment during the use of TenolamE or N does not contraindicate the prescription of Tenofovir but the pts’ renal function should be monitored regularly	N/A
Kamkuemah: 2015	The incidence of decline in renal function of > 10 ml/min/1.73 m^2^ in 12 months after ART initiation was 96 per 100 person-years. This incidence was greatest during the first 2 months on ART (208 per 100 person-years). Overall, 3% of patients experienced declines in renal function below 50 ml/min/1.73 m^2^ over 12 months (with seven people detected at month 1, 2 people detected at month 2 and 1 detected at month 4)	Tenofovir can be administered safely in primary health care after the initial pre-ART screening of creatinine clearance, to identify high-risk cases. Renal function generally improves in parallel with other health improvements on ART. Benefits of tenofovir initiation outweigh negative effects it might have, at least during the first 12 months of use	N/A
Mayanja: 2017	Among individuals on long-term ART; there were no differences in renal dysfunction (glomerular function and renal tubular function) between patients on Tenofovir containing and Non-Tenofovir containing ART regimens	Tenofovir based first line ART can safely be initiated even in settings without routine renal function monitoring	N/A
Mpondo: 2014	At the time of follow-up, patients’ eGFRs by CG equation had improved, from a median of 74.1 (55.8–100.1) at baseline to 103.4 (85.3–135.6) at follow-up (p 0.001) (Table 1). At follow-up, 36 of 171 (21.1%) had decreased eGFRs of, 90, and only 2 (1.2%) had eGFRs, 60 compared to 107/171 (62.6%) and 36/171 (21.1%) respectively at baseline (p 0.001 for each). By MDRD, 23 of 171 (13.5%) had eGFRs, 90, and the same two patients had eGFRs, 60. The prevalence of microalbuminuria decreased from 72.1 to 43.9% (p 0.001), and the prevalence of proteinuria decreased from 35.7 to 8.8% (p 0.001) at follow-up. All eight patients who had had initial eGFRs, 60 and were treated with tenofovir had follow-up eGFRs. 60 and stable (n = 3) or improved (n = 5) microalbuminuria and proteinuria	Safe	N/A
Reid: 2008	Up to 96 weeks, the incidence of severe renal impairment was no different between tenofovir DF containing and other ART regimens. Overall, 11 (0.3%) participants died with renal disease contributing to their death; however, these 11 deaths represented only 5% of all deaths to 96 weeks. Despite a relatively high baseline prevalence of mild-to-moderate renal dysfunction in African adults with low CD4+ cell counts, severe eGFR impairment after ART initiation was infrequent with all regimens	Patients have increased risk of reduced eGFR but no increased risk of renal failure	N/A
Salome: 2016	Mean eGFR lower among TDF-ART grp than in non-TDF ART grp (p = 0.001)using CKI-Epi and MDRD formula with race adjustments (p = 0.008) but no differences using CG. Using all formulae, although proportions of participants with abnormal eGFR < 60 were higher among the TDF-ART grp than the non, the differences were insignificant. No sig differences in the adjusted mean differences of eGFR when diff durations on different TDF exposure ART regimens were compared. No differences in fractional tubular phosphate reabsorption	Safe	N/A
Stöhr: 2011	At ART initiation, median CD4+ T cell count was 86 cells/mm^3^; 1492 (45%) participants had mild (60 to < 90 ml/min/1.73 m^2^), 237 (7%) moderate (30 to < 60 ml/min/1.73 m^2^) and 7 (0.2%) severe (15 to < 30 ml/min/1.73 m^2^) decreases in eGFR. First-line ART was zidovudine/lamivudine plus tenofovir (74%), abacavir (9%) or nevirapine (17%). By 4 years, cumulative incidence of eGFR < 30 ml/min/1.73 m^2^ was 2.8% (n = 90) and CKD was 5.0% (n = 162). Adjusted eGFR increases to 4 years were 1, 9 and 6 ml/min/1.73 m^2^ with tenofovir, abacavir and nevirapine, respectively (p < 0.001), and 4 and 2 ml/min/1.73 m^2^ for laboratory and clinical monitoring, respectively (p = 0.005; 2 and 3 ml/min/1.73 m^2^ to 5 years; p = 0.81)	Safe	N/A
Shamu: 2015	Incidence of nephropathy was low in this cohort	Safe	N/A
Tewogbade: 2010	The creatinine clearance (MDRD) improved from 47.9 ± 18.42 ml/min/1.73 m^2^ for patients before treatment to 57.9 ± 9.43 ml/min/1.73 m^2^ at 9 months. The eGFR by Cockcroft-Gault did not show any statistically significant difference between pre-treatment and 9 months post treatment values The plasma creatinine also improved significantly from the pre-treatment value of 131.1 μmol/L to 93.4 μmol/L at 9 months but two patients values increased from 346 and 44 μmol/L to 707 and 563 μmol/L	Desirable safety	N/A
Zannou: 2015	The prevalence of chronic kidney failure is relatively high (18.7%) in this study. Tenofovir was used by 12.9% of patients in this cohort and was not associated with the occurrence of chronic kidney failure	Safe	N/A
Cournil: 2016	Initiation of PI/r-based second-line regimen induced a marked eGFR decline of − 10.5 ml/min/1.73 m^2^ at week 4 in all treatment groups with a greater decrease in TDF/FTC + LPV/r arm (− 15.1 ml/min/1.73 m^2^). At month 18, mean eGFR in the non-TDF containing regimen recovered its baseline level and was significantly greater than eGFR 18-month levels in the TDF-containing regimens that experienced only partial recovery (difference: − 10.7; CI − 16.8, − 4.6; p = 0.001 in TDF/FTC + LPV/r and − 6.4; CI − 12.5, − 0.3; p = 0.04 in TDF/FTC + DRV/r). At 18 months, prevalence of stage 3 chronic kidney disease was low (< 3%) and not associated with treatment. One treatment discontinuation and five TDF dosage reductions for renal toxicities were reported in TDF-containing arms	These results suggest a reasonable renal tolerance of a regimen associating TDF/FTC + PI/r in African patients with eGFR > 60 ml/ml/1.73 m (2) at baseline	Recommend reassessment of renal function 1 month after initiation of treatment including ritonavir
Gajee: 2016	The CrCl in the younger age group (≥ 20 to < 30 years) exhibited an increase in CrCl at 12 months post-TDF commencement. The older age group (≥ 30 to ≤ 40 years) displayed a decrease in CrCl at 12 months post-TDF commencement for females and males	Safe	Age and gender influence kidney function
Myer: 2013	The median serum creatinine in pregnant women (46 mmol/L) was significantly lower and the median creatinine clearance (163 ml/min/1.73 m^2^) was significantly higher than other groups (p < 0.001 and p = 0.004, respectively). Fewer than 1% of pregnant women had moderate renal dysfunction before ART initiation, with no instances of severe dysfunction observed, compared to 7% moderate or severe renal dysfunction in non-pregnant women or men (P < 0.001)	Renal dysfunction in HIV-infected pregnant women is significantly less common than in other HIV-infected adults eligible for ART. The risks associated with initiating tenofovir immediately in pregnant women before reviewing serum creatinine results may be limited, and the benefits of rapid ART initiation in pregnancy may outweigh possible risks of nephrotoxicity	N/A
Chadwick, D. R:2015	Pts on TDF had significantly higher uPCRs (10.8 vs 5.7 mg/mmol, p < 0.001) and lower uAPRs (0.24 vs 0.58, p < 0.001). 35% of those on TDF (vs 6% not on TDF)satisfied the criteria for TD	Both proteinuria and TD are common and associated with TDF use in Ghana. TDF significantly independently associated with TD and proteinuria though no clinically significant TD found	No clinical significance found
Agbaji: 2011	sCr in TDF↑18% and in Non TDF↑1.2% b) TDF GFR = ↓4.8% Non TDF↑5.1%	TDF statistically associated with decline in CrCl	Slight
Brennan: 2011	At initiation, 64.4% had normal (> 90 ml/min) renal function, 30.4% had mild (60–90 ml/min) RD, and 5.2% had moderate (30-59 ml/min) RD. After 48 months 21 (2.4%) experienced nephrotoxicity, 6 of these died from it	Pre-existing renal pathology may be exacerbated by TDF	Significant in pts with pre-existing renal pathology
Bygrave: 2011	Among 933 adults for whom baseline creatinine was available, 176 (18.9%) presented with a baseline CrCl < 50 ml/min. Renal function improved during follow-up. 19 patients who developed renal toxicity during follow up remained on TDF; renal function improved (CrCl ≥ 50 ml/min) in all but 3 of these patients. Among 15 patients with a baseline CrCl < 50 ml/min were started in error, none developed severe renal impairment	Rare and transient	Rare and transient
Dekert: 2017	TDF pts had a median eGFR (unadjusted) decrease of − 6.5, TDF-free, − 3.0 and ART switched − 8.5 l/min/1.73 m^2^ over the course of the observation. Three distinct developments observed: (1) some persons with initially normal or mildly impairment eGFR lost renal filtration over time, in addition to the aging effect (2) persons whose renal function was initially severely impaired regained renal filtration most likely because of viral clearance (3) a subgroup of individuals who were identified with impaired renal function deteriorated or remained at a low level despite switching to a TDF free regimen	Individuals always receiving TDF showed only a slight but not significant eGFR reduction	Slight/not significant
Mugomeri, E:2014	In 56 patients (17.9%), TDF was found to be contraindicated. The use of TDF was marginally significant factor for renal toxicity (p 0.054) in univariate analysis, but was insignificant (p 0.122) in multivariate logistic analysis	TDF a weak contributing factor of renal impairment.	Weak. Routine baseline renal function screening should be adopted to prevent patients with impaired renal function receiving TDF
Mulenga: 2014	For the outcome defined as incident episodes of moderate or severe eGFR decrease, the differences reached statistical significance; however, the numbers remained low (1.90% in the TDF vs 1.27% in the non TDF group [P < 0.001] at 6 months and 1.84% vs 1.37% [P = 0.02] at 12 months	Patients receiving TDF were more likely to experience an episode of moderate or severe renal dysfunction than those receiving other regimens during the first year of ART	Rare
Ndagije: 2015	There was one suspected renal toxicity reported for every 200 patients on a tenofovir-based regimen. Some of the serious reactions reported were death in two cases and bone demineralisation in five patients. Hose that had been on tenofovir for more than 4 years had raised serum creatinine levels, emphasising the importance of monitoring for the risk of renal damage	Occurrence of suspected tenofovir renal toxicity of HIV patients is low, there is need to monitor those at risk so as to prevent irreversible kidney injury	Low. Need to monitor those at risk so as to prevent irreversible kidney injury
De Beaudrap: 2010	Between 1–12 months, pts on TDF experienced a↑ of transition from mild renal impairment (60–90 ml/min/1.73 m^2^) to moderate (30–60 ml/min/1.73 m^2^) compared with pts not receiving TDF who experienced an ↑ of 4.33 ml/min but change was not significant. For pts on TDF significant ↓ of − 10.4 ml/min observed. After 12 months eGFR remained stable for the non TDF pts but ↓ from month12-42 by − 4.1 ml/min/1.73 m^2^. Proportion of pts on TDF with moderate impairment ↑ from 8.6% at month 12 to 20% at month 42. No↓ in eGFR below 30 ml/min/1.73 m^2^	TDF associated with significant but moderate decline in RD. Consequent impairment persistent after 1st year of treatment	Moderate
Mulubwa: 2016	No significant correlations were found between plasma TFV concentration and eGFR, CrCl, TmPO4/GFR, SCr, UNa, serum urea or serum uric acid (p > 0.05). Nevertheless, a positive correlation was found between TFV plasma concentration and albuminuria (unadjusted r = 0.606; p = 0.001. TFV concentration was independently associated with increased albuminuria	Plasma TFV concentration is independently associated with increased albuminuria in HIV-infected women within this pilot investigation. There was an increase in eGFR and CrCl in the HIV-infected women from baseline	Moderate
De Waal: 2017	Amongst those with a baseline and subsequent eGFR available, mean eGFR change from baseline at 12 months was − 4.4 mL/min (95% CI − 4.9 to − 4.0), − 2.3 (− 2.5 to − 2.1), and 0.6 (0.04 to 1.2) in those with baseline eGFR 90 mL/min; and 11.9 mL/min (11.0 to 12.7), 14.6 (13.5 to 15.7), and 11.0 (10.3 to 11.7) in those with baseline eGFR < 90 mL/min, according to the MDRD, CKD-EPI (n = 11 112), and Cockcroft-Gault (n = 9 283) equations, respectively. Overall, 292 (1.9%) patients developed eGFR < 30 mL/min. Significant associations with low eGFR included older age, baseline eGFR < 60 mL/min, CD4 count < 200 cells/μL, body weight < 60 kg, and concomitant protease inhibitor use	Overall pts on TDF experienced declines in eGFR over time. In the subgroup of pts who had moderate or severe kidney dysfunction at baseline, eGFR improved substantially on treatment regardless of equation used	Small but significant in pts with normal baseline eGFR
Orluwene: 2015	The differences between the values were not statistically significant as observed in the TDF group. In this study estimated glomerular filtration rate (eGFR) increased only slightly at 12 weeks of exposure particularly in the TDF regimen group pointing a delay in the detection of proximal tubular dysfunction compared to IL-18 that shows a marked increased at 4 weeks. There was also an increase in IL-18 levels with time, suggesting a possible progression of renal dysfunction from a subclinical stage to an end stage renal disease	there might exist a possible relationship between nephrotoxicity caused by TDF and increased in IL-18 levels in HIV-infected patients on TDF first line ART	Highly statistically significant
Seedat: 2017	61% of TDF grp had AKI on admission vs 43%. Discharge median sCr was higher in the TDF group and fewer in the TDF group recovered renal function after 3 months	TDF exposed HIV-infected patients who develop AKI have a similar etiology, rate & range of nephrotoxic risk factors as those not receiving TDF. However, data suggest TDF has an added nephrotoxic effect in patients with AKI causing: a more rapid worsening of renal function; a higher proportion with proteinuria and acidosis; and delayed renal recovery	Notably high
Wantakisha; 2017	Point prevalence of renal dysfunction among HIV-positive adults exposed to TDF was 18.6% at 18 months follow up. Patients with a CD4 + cell count > 350 cells/uL had decreased odds of developing renal dysfunction by 81% and this decrease could be as low as 79% to as high as 97% adjusting for other covariates	Renal dysfunction was concentrated in older patients with low CD4 + cell count. Thus, close renal monitoring in these patients when initiating TDF-based treatment should be intensified	High burden
Zachor: 2016	55% experienced RKFD, and 2% developed stage 3 CKD. For every 10 y increase in age and 10 ml/min lower baseline eGFR, the odds of RFKD increased by 70%. Each 10y older age was associated with a 1.9 fold increased risk of developing stage 3 CKD. Women had a 4 fold greater risk of stage 3 CKD than men	TDF associated with both a higher likelihood of RKFD and stage 3 CKD among HIV infected South Africans	High burden
Mwafongo: 2014	3.2% experienced renal events.2% required permanent treatment modification. Events primarily occurred early after starting treatment with a decreasing rate over time	The primary events involving the use of TDF in RSL were uncommon (3%) thereby limiting the power to evaluate possible factors associated with risk	Could not assess

## Discussion

**Fig. 2:**
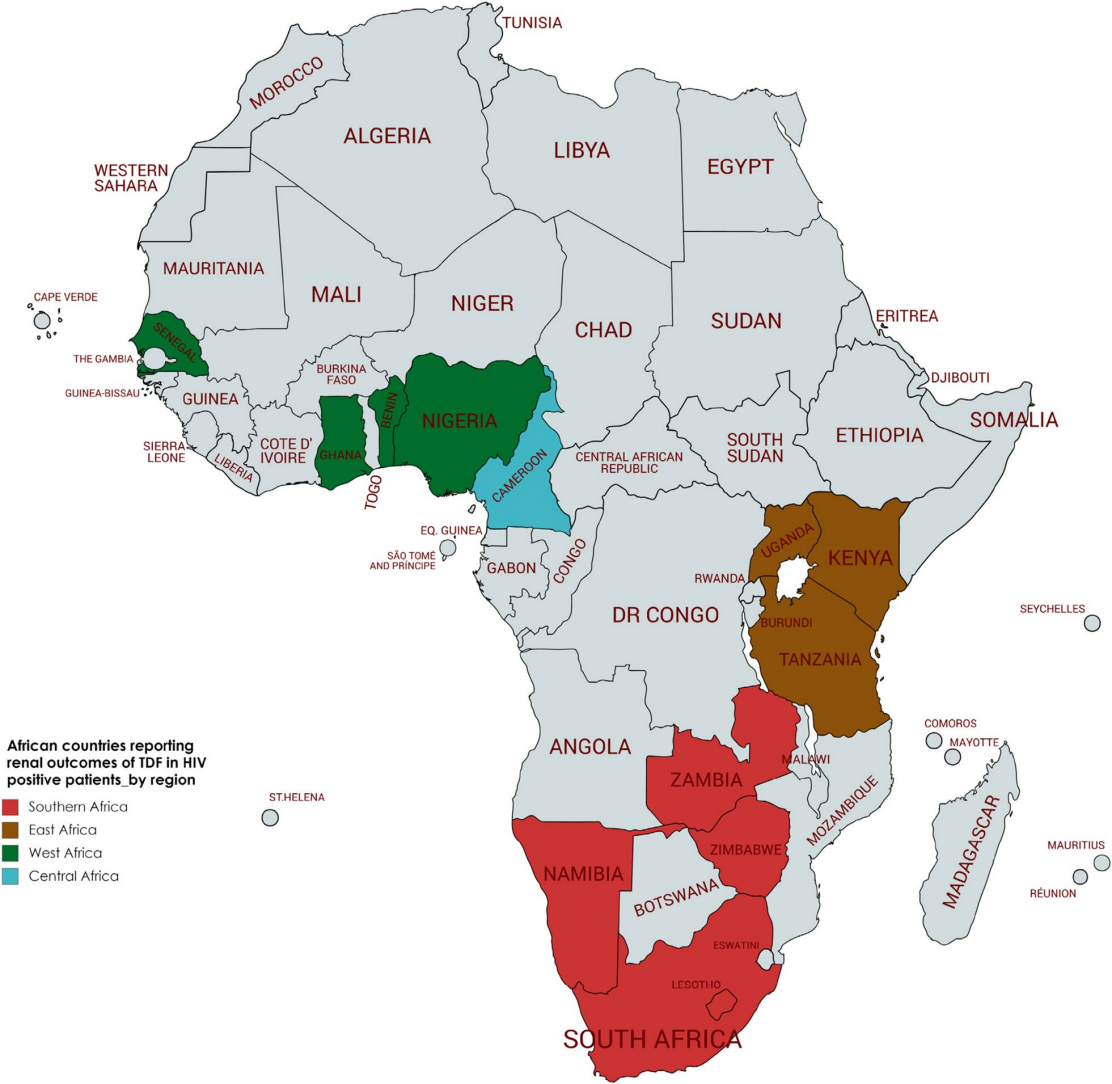
African countries reporting renal outcomes of HIV positive patients on a Tenofovir-containing regimen

Our review is the first to systematically document renal outcomes of patients on tenofovir-containing ART in African populations. We identified 31 eligible studies involving a total of 106, 406 participants in 14 countries. These included five countries of Southern Africa, five from West Africa, three from East Africa and one from Central Africa (Fig. 2).

The results (summarised in Table 3) indicate conflicting evidence of the association of TDF with renal dysfunction. During follow up terms of up to 9 years, 50% of the studies suggest overall safety of tenofovir while the other half report varying levels of renal toxicity due to TDF. Fifteen studies reported that there is neither statistical nor clinical association of TDF with renal function decline in HIV-positive patients [27–31, 36–40, 42, 44, 46, 48–50]. Although some recommend monitoring of patients particularly those with deranged renal function parameters at baseline, there is, no consensus on recommended monitoring strategies to this end. On the other hand, 16 studies reported an association with seven of these reporting the association as significant (Table 3) [15, 22–26, 32–35, 43, 45, 47, 51–53]. Five of the seven were done in Southern Africa [25, 34, 35, 43, 45]. One study could not make an assessment of whether or not TDF was associated with renal function decline in these patients [47]. Unfortunately, of the studies included in this review, only four systematically documented other non-ART co-medications that these patients were taking thereby making it difficult to make assessments of the impact of other drugs on the results or to conclusively attribute the impact on renal function of TDF containing ART of the studies in this review [25, 26, 28, 47]. However, Cournil et al. suggest that an interaction between Tenofovir and Ritonavir may be responsible for an initial decrease in eGFR observed when a patients initiates on a ritonavir boosted TDF regimen. In a recent review by Hill et al. the authors support this finding that TDF toxicity occurs when it is used with boosting agents such at ritonavir [54]. This is important in cases where Protease inhibitor (PI) boosted regimens are required in second and third line therapy. In ageing HIV populations with increased lifespans, second and third line therapy is particularly important and close monitoring of these patients cannot be over-emphasized.

The mean duration on TDF treatment of the studies was less than 3 years in most studies with two studies both from Uganda reporting outcomes in patients who had taken the drug for a median of 9 years as a drug that can be safely given without serious renal implications [30, 44]. The Zambian study reported by Watankisha found a high burden of renal dysfunction in older patients with low CD4 counts reporting point prevalence of kidney dysfunction among these patients as 18.6% at 18 months follow up [45].

There was no consistency in the outcome being measured (Table 1). More importantly, while it is understood that TDF specifically affects proximal tubular dysfunction, the studies in this review generally did not systematically use tests specific for proximal tubular dysfunction such as Fanconi syndrome [41, 55, 56].

### Safety of TDF

With ageing of the HIV populations in the era of ART, long-term complications of HIV such as renal disease become important. However, there are still limited drug safety data on tenofovir-associated renal dysfunction in adolescents and children. In this review, the studies reported were performed in adults mean age ≥ 33 years; only one included patients below 16 years, being pregnant females aged 13 and older, the physiology of whom is not representative of other adolescents. As such, these results may not generalizable to the younger populations who are in fact dosed the same as adults as long as they are > 35 kg or 10 years or older and will potentially take tenofovir for longer. It follows, therefore, that importance should be placed on monitoring the younger patients on TDF to potentially reduce co-morbidities with non-communicable diseases (NCDs) such as chronic kidney disease and also the potential burden of polypharmacy resulting later in life. This is especially important in the context of already strained African health systems. Given the recommendation and adoption of the test and treat approach and the fact that tenofovir is still in widespread use in African resource limited countries, it is imperative to further investigate the cumulative risk for TDF-associated nephrotoxicity during prolonged use in patients initiating its use early in their lives.

This review also notes that there is still a gap in knowledge of the impact of TDF in pregnant women against the background that TDF containing regimens are still the backbone of Prevention of Mother to Child Transmission (PMTCT) programmes in Africa. The three studies that focused on pregnant women all reported different outcomes: Myer reported that the risk of TDF associated toxicity is reduced in pregnant women, Mulubwa suggested that the risk is moderately higher in pregnant women while Mwafongo could not assess due to the small sample size [36, 43, 47].

Our review is the only such review done exclusively on Africans focusing on Tenofovir associated renal toxicity. A similar review had been conducted by Cooper et al. in 2012 but on high-income countries essentially excluding African countries. None of the studies found in this review focused on children or adolescents. In the review by Cooper, it was recommended that future trials focus on resource limited settings. It was noted in the present review that there has, indeed, been an increase in similar trials in resource limited settings since then [11]. Despite including patients of different ancestry, our results are in agreement with Cooper’s review i.e. although relatively low and modest, there exists an association between TDF and renal decline. Several studies on different populations have investigated renal transporter single nucleotide polymorphisms (SNPs) related to possible TDF renal toxicity with conflicting results [57–62]. It may be important to do similar studies on patients of African ancestry in order to determine its contribution to the discussion and if found, the consequent effect size. The results observed in the various geographical areas within Africa, where the studies included in this review were performed, do not show any particular pattern in the outcomes, associated with any region.

### Future areas for developing research

As mentioned in the 2016 WHO guidelines, further investigations are still required on the long term safety of tenofovir disoproxil fumarate as more data are needed on bone, growth and renal toxicity profiles in adolescents and children. More data are still needed to understand the possibility, impact and clinical implications of TDF toxicity in adolescents. Since Southern Africa seemed to report less desirable renal safety profiles of TDF in HIV positive patients, pharmacogenomic screening for specific genetic markers predisposing patients to renal disease may also be beneficial in identifying patients at higher risk of developing CKD in the different African populations.

Given that several studies have suggested a need for routine monitoring of patients on TDF, there is also still a gap in the systematic comparison and documentation of monitoring strategies that may be effective in these settings.

### Limitations of current study

The study was limited by the available literature; it is possible that more studies may have been done in Africa but were not published by the time of this review. The heterogeneity in the definitions of renal outcomes and how they were reported had a negative impact on our pooled analysis of study results and the inability to perform an effective meta-analysis. Too few studies reported specific markers of proximal tubulopathy. While policies are clear on what needs to be checked for TDF induced nephropathy, there needs to be a better documentation on how best to report these in clinical practice to allow for more effective comparisons. There were only a few studies that reported concomitant ART regimens so this review could not assess the possibility of other ART as possible effect modifiers of TDF-associated nephrotoxicity. It is also important to note that the present review could not analyse for the effect of other diseases such as hypertension and diabetes on renal dysfunction as they were not systematically reported.

All studies were essentially conducted in adults; future research should focus on younger populations as the long term implications of HIV and ART become of greater cause for concern with an ageing HIV population on continued ART.

## Conclusions

Our review identified studies in Africans reporting statistically significant renal function decline associated with TDF use but the clinical significance of this effect was not enough to contraindicate its continued use in ART regimens (Table 3). Consistent with studies in other populations, patients are at greater risk if they have pre-existing renal disease and are more advanced in age. However, more long term research is required (studying cohorts over at least a decade) that monitors clinically relevant markers specific for proximal tubulopathy. This is particularly important for younger, paediatric populations whose life expectancy has improved as they are potentially on ART for decades from childhood but are also at increased risk of developing NCDs such as diabetes, CVD and cancers which could also influence renal toxicity.

## Additional file

**Additional file 1:** Search strategy for Pubmed (as at 5 October 2017).

## Data Availability

The datasets during and/or analysed during the current study available from the corresponding author on reasonable request.

## Abbreviations

AKI: acute kidney injury
ART: antiretroviral treatment
CKD: chronic kidney disease
CKI-EPI: chronic kidney disease epidemiology collaboration
CrCl: creatinine clearance
D4T: stavudine
EFV: efavirenz
eGFR: estimated glomerular filtration rate
FTC: emtricitabine
HIV: human immunodeficiency virus
IL: interleukin
LPV: lopinavir
MDRD: modification of diet in renal disease
NVP: nevirapine
PMTCT: prevention of mother to child transmission
RCT: randomised control trial
RD: renal dysfunction
sCr: serum creatinine
SNP: single nucleotide polymorphism
TD: tubular dysfunction
TDF: tenofovir disproxil fumarate
TAF: tenofovir alafenamide

## References

[1] Wishart DS, Knox C, Guo AC, Cheng D, Shrivastava S, Tzur D (2008). DrugBank: a knowledgebase for drugs, drug actions and drug targets. Nucleic Acids Res.

[2] Knox C, Law V, Jewison T, Liu P, Ly S, Frolkis A (2011). DrugBank 3.0: a comprehensive resource for ‘omics’ research on drugs. Nucleic Acids Res.

[3] da Rocha IM, Gasparotto AS, Lazzaretti RK, Notti RK, Sprinz E, Mattevi VS (2015). Polymorphisms associated with renal adverse effects of antiretroviral therapy in a Southern Brazilian HIV cohort. Pharmacogenet Genom.

[4] Kohler JJ, Hosseini SH, Green E, Abuin A, Ludaway T, Russ R (2011). Tenofovir renal proximal tubular toxicity is regulated By OAT1 and MRP4 transporters. Lab Invest.

[5] Pushpakom SP, Liptrott NJ, Rodríguez-Nóvoa S, Labarga P, Soriano V, Albalater M (2011). Genetic variants of ABCC10, a novel tenofovir transporter, are associated with kidney tubular dysfunction. J Infect Dis.

[6] Rodriguez-Novoa S, Labarga P, Soriano V (2009). Pharmacogenetics of tenofovir treatment. Pharmacogenomics..

[7] Rodriguez-Novoa S, Labarga P, Soriano V, Egan D, Albalater M, Morello J (2009). Predictors of kidney tubular dysfunction in HIV-infected patients treated with tenofovir: a pharmacogenetic study. Clin Infect Dis.

[8] Coca S, Perazella MA (2002). Rapid communication: acute renal failure associated with tenofovir: evidence of drug-induced nephrotoxicity. Am J Med Sci.

[9] Fernandez-Fernandez B, Montoya-Ferrer A, Sanz AB, Sanchez-Niño MD, Izquierdo MC, Poveda J (2011). Tenofovir nephrotoxicity: 2011 update. AIDS Res Treat.

[10] Nelson MR, Katlama C, Montaner JS, Cooper DA, Gazzard B, Clotet B (2007). The safety of tenofovir disoproxil fumarate for the treatment of HIV infection in adults: the first 4 years. AIDS (London, England)..

[11] Cooper RD, Wiebe N, Smith N, Keiser P, Naicker S, Tonelli M (2010). Systematic review and meta-analysis: renal safety of tenofovir disoproxil fumarate in HIV-infected patients. Clin Infect Dis.

[12] Fabian J, Naicker S (2009). HIV and kidney disease in sub-Saharan Africa. Nat Rev Nephrol.

[13] Lucas GM, Lau B, Atta MG, Fine DM, Keruly J, Moore RD (2008). Chronic kidney disease incidence, and progression to end-stage renal disease, in HIV-infected individuals: a tale of two races. J Infect Dis.

[14] Kalyesubula R, Perazella MA (2011). Nephrotoxicity of HAART. AIDS Res Treat.

[15] Brennan A, Evans D, Maskew M, Naicker S, Ive P, Sanne I (2011). Relationship between renal dysfunction, nephrotoxicity and death among HIV adults on tenofovir. AIDS (London, England)..

[16] Langness JA, Hindman JT, Johnson SC, Kiser JJ (2013). The frequency of adjusted renal dosing of tenofovir DF and its effects on patient outcomes. J Pharm Pract.

[17] Gupta SK (2005). Tenofovir and changes in renal function. Clin Infect Dis.

[18] Sax PE, Gallant JE, Klotman PE (2007). Renal safety of tenofovir disoproxil fumarate. AIDS Reader..

[19] Wang H, Lu X, Yang X, Xu N (2016). The efficacy and safety of tenofovir alafenamide versus tenofovir disoproxil fumarate in antiretroviral regimens for HIV-1 therapy: meta-analysis. Medicine..

[20] Moher D, Liberati A, Tetzlaff J, Altman DG, The PG (2009). Preferred reporting items for systematic reviews and meta-analyses: the PRISMA statement. PLoS Med.

[21] Masese LA, Okalebo FA, Mwangangi LEM, Bosire KO, Mwangi M (2012). Comparison of the renal safety of tenofovir and stavudine in patients on antiretroviral therapy at a kenyan referral hospital. Pharmacoepidemiol Drug Saf.

[22] Agbaji OO, Agaba PA, Idoko JA, Taiwo B, Murphy R, Kanki P (2011). Temporal changes in renal glomerular function associated with the use of tenofovir disoproxil fumarate in HIV-infected Nigerians. West Afr J Med.

[23] Beaudrap PD, Diallo MB, Landman R, Gueye NF, Ndiaye I, Diouf A (2010). Changes in the renal function after tenofovir-containing antiretroviral therapy initiation in a Senegalese cohort (ANRS 1215). AIDS Res Hum Retroviruses.

[24] Bygrave H, Kranzer K, Hilderbrand K, Jouquet G, Goemaere E, Vlahakis N (2011). Renal safety of a tenofovir-containing first line regimen: experience from an antiretroviral cohort in rural lesotho. PLoS ONE.

[25] De Waal R, Cohen K, Fox MP, Stinson K, Maartens G, Boulle A (2017). Changes in estimated glomerular filtration rate over time in South African HIV-1-infected patients receiving tenofovir: a retrospective cohort study. J Int AIDS Soc.

[26] Deckert A, Neuhann F, Klose C, Bruckner T, Beiersmann C, Haloka J (2017). Assessment of renal function in routine care of people living with HIV on ART in a resource-limited setting in urban Zambia. PLoS ONE.

[27] Hema A, Cournil A, Ciaffi L, Eymard-Duvernay s, Diouf a, Manga n (2015). Impact of TDF + PI/R on renal function in Sub-Saharan Africa: 2LADY/ANRS 12169 trial. Topics Antiviral Med.

[28] Kalemeera F, Mbango C, Mubita M, Naikaku E, Gaida R, Godman B (2016). Effect of changing from first- to second-line antiretroviral therapy on renal function: a retrospective study based on data from a single health facility in Namibia. Exp Rev Anti-Infect Ther.

[29] Kamkuemah M, Kaplan R, Bekker LG, Little F, Myer L (2015). Renal impairment in HIV-infected patients initiating tenofovir-containing antiretroviral therapy regimens in a Primary Healthcare Setting in South Africa. Trop Med Int Health.

[30] Mayanja BN, Kasamba I, Levin J, Namakoola I, Kazooba P, Were J (2017). COHORT PROFILE: the complications of long-term antiretroviral therapy study in Uganda (CoLTART), a prospective clinical cohort. AIDS Res Ther.

[31] Mpondo BCT, Kalluvya SE, Peck RN, Kabangila R, Kidenya BR, Ephraim L (2014). Impact of antiretroviral therapy on renal function among HIV-infected Tanzanian adults: a retrospective cohort study. PLoS ONE.

[32] Mugomeri E, Olivier D, Van Den Heever-Kriek E (2014). The effect of tenofovir in renal function in HIV-positive adult patients in the Roma health service area, Lesotho, southern Africa. J Int AIDS Soc.

[33] Mulenga L, Musonda P, Mwango A, Vinikoor MJ, Davies MA, Mweemba A (2014). Effect of baseline renal function on tenofovir-containing antiretroviral therapy outcomes in Zambia. Clin Infect Dis.

[34] Seedat F, Martinson N, Motlhaoleng K, Abraham P, Mancama D, Naicker S (2017). Acute kidney injury, risk factors, and prognosis in hospitalized HIV-infected adults in South Africa, compared by tenofovir exposure. AIDS Res Hum Retroviruses.

[35] Zachor H, Machekano R, Estrella MM, Veldkamp PJ, Zeier MD, Uthman OA (2016). Incidence of stage 3 chronic kidney disease and progression on tenofovir-based regimens. AIDS (London, England)..

[36] Myer L, Kamkuemah M, Kaplan R, Bekker LG (2013). Low prevalence of renal dysfunction in HIV-infected pregnant women: implications for guidelines for the prevention of mother-to-child transmission of HIV. Trop Med Int Health.

[37] Shamu T, Wellington M, Pascoe M, Gwanzura L, Ndhlovu C (2015). Incidence of nephropathy in HIV infected patients receiving highly active antiretroviral therapy at Newlands Clinic: a retrospective study. World J AIDS.

[38] Gajee AA. The renal safety profile of tenofovir as used in combination antiretroviral therapy: North-West University (South Africa), Potchefstroom Campus; 2016.

[39] Tewogbade AA (2010). Effects of highly active antiretroviral therapy haart on the renal function of HIV/AIDS patients at the university of Ilorin Teaching Hospital, Ilorin.

[40] Banda J, Mweemba A, Siziya S, Mweene M, Andrews B, Lakhi S (2010). Prevalence and factors associated with renal dysfunction in HIV positive and negative adults at the University Teaching Hospital, Lusaka. Med J Zambia.

[41] Chadwick DR, Sarfo FS, Kirk ES, Owusu D, Bedu-Addo G, Parris V (2015). Tenofovir is associated with increased tubular proteinuria and asymptomatic renal tubular dysfunction in Ghana. BMC Nephrol.

[42] Fritzsche C, Rudolph J, Huenten-Kirsch B, Hemmer CJ, Tekoh R, Kuwoh PB (2017). Effect of tenofovor diproxil fumarate on renal function and urinalysis abnormalities in HIV-infected Cameroonian adults. Am J Trop Med Hyg.

[43] Mulubwa M, Rheeders M, Fourie C, Viljoen M (2016). Associations between plasma tenofovir concentration and renal function markers in HIV-infected women. S Afr J HIV Med.

[44] Salome T, Kasamba I, Mayanja BN, Kazooba P, Were J, Kaleebu P (2016). The effect of Tenofovir on renal function among Ugandan adults on long-term antiretroviral therapy: a cross-sectional enrolment analysis. AIDS Res Ther.

[45] Wantakisha E, Chongwe G, Munkombwe D, Michelo C (2017). Renal dysfunction among HIV-infected patients on tenofovir-based antiretroviral therapy at Ronald Ross Hospital in Zambia. J AIDS Clin Res.

[46] Zannou D, Vigan J, Azon-Kouanou A, Agboton B, Houngbe C (2015). Prevalence of chronic kidney failure and associated factors in patients treated by antiretroviral in the national teaching hospital of Cotonou. J Nephrol Ther.

[47] Mwafongo A, Nkanaunena K, Zheng Y, Hogg E, Samaneka W, Mulenga L (2014). Renal events among women treated with tenofovir/emtricitabine in combination with either lopinavir/ritonavir or nevirapine. AIDS (London, England)..

[48] Cournil A, Hema A, Eymard-Duvernay S, Ciaffi L, Badiou S, Kabore FN (2017). Evolution of renal function in African patients initiating second-line antiretroviral treatment: findings from the ANRS 12169 2LADY trial. Antiviral Ther.

[49] Reid A, Stöhr W, Walker AS, Williams IG, Kityo C, Hughes P (2008). Severe renal dysfunction and risk factors associated with renal impairment in HIV-infected adults in Africa initiating antiretroviral therapy. Clin Infect Dis.

[50] Stöhr W, Reid A, Walker AS, Ssali F, Munderi P, Mambule I (2011). Glomerular dysfunction and associated risk factors over 4-5 years following antiretroviral therapy initiation in Africa. Antiviral Ther.

[51] Orluwene CG, Deebii N, Odum EP (2015). Urinary interleukin (Il)-18 as an early predictive biomarker of subclinical proximal tubular dysfunction in HIV-infected patients exposed to tenofovir. J AIDS Clin Res.

[52] Ndagije H, Nambasa V, Namagala E, Nassali H, Kajungu D, Sematiko G (2015). Targeted spontaneous reporting of suspected renal toxicity in patients undergoing highly active anti-retroviral therapy in two public health facilities in Uganda. Drug Saf.

[53] Chadwick, Sarfo FS, Kirk ESM, Owusu D, Bedu-Addo G, Parris V (2015). Tenofovir is associated with increased tubular proteinuria and asymptomatic renal tubular dysfunction in Ghana. BMC Nephrol.

[54] Hill A, Hughes SL, Gotham D, Pozniak AL (2018). Tenofovir alafenamide versus tenofovir disoproxil fumarate: is there a true difference in efficacy and safety?. J Virus Erad.

[55] Andrade-Fuentes K, Mata-Marin JA, Lopez-De Leon JI, Manjarrez-Tellez B, Ramirez JL, Gaytan-Martinez J (2015). Proximal renal tubular dysfunction related to antiretroviral therapy among HIV-infected patients in an HIV clinic in Mexico. AIDS Patient Care STDs.

[56] Bonjoch A, Echeverria P, Perez-Alvarez N, Puig J, Estany C, Clotet B (2016). Prospective study to assess progression of renal markers after interruption of tenofovir due to nephrotoxicity. Biomed Res Int.

[57] Arruda MB, Campagnari F, de Almeida TB, Couto-Fernandez JC, Tanuri A, Cardoso CC (2016). Single nucleotide polymorphisms in cellular drug transporters are associated with intolerance to antiretroviral therapy in Brazilian HIV-1 positive individuals. PLoS ONE.

[58] Cheong HS, Kim HD, Na HS, Kim JO, Kim LH, Kim SH (2011). Screening of genetic variations of SLC15A2, SLC22A1, SLC22A2 and SLC22A6 genes. J Hum Genet.

[59] Limou S, Nelson GW, Kopp JB, Winkler CA (2014). APOL1 kidney risk alleles: population genetics and disease associations. Adv Chronic Kidney Dis..

[60] Manosuthi W, Sukasem C, Thongyen S, Nilkamhang S, Sungkanuparph S (2014). ABCC2*1C and plasma tenofovir concentration are correlated to decreased glomerular filtration rate in patients receiving a tenofovir-containing antiretroviral regimen. J Antimicrob Chemother.

[61] Nishijima T, Hayashida T, Kurosawa T, Tanaka N, Oka S, Gatanaga H (2015). Drug transporter genetic variants are not associated with TDF-related renal dysfunction in patients with HIV-1 infection: a pharmacogenetic study. PLoS ONE.

[62] Rungtivasuwan K, Avihingsanon A, Thammajaruk N, Mitruk S, Burger DM, Ruxrungtham K (2015). Influence of <em>ABCC2</em> and <em>ABCC4</em> polymorphisms on tenofovir plasma concentrations in Thai HIV-infected patients. Antimicrob Agents Chemother.

