# Supplementary material for: Tenofovir-associated kidney disease in Africans: a systematic review
Source: AIDS Res Ther. 2019 Jun 6;16:12. doi: 10.1186/s12981-019-0227-1 (PMC6554992; doi:10.1186/s12981-019-0227-1)
Supplement: Supplementary file 1 — Additional file 1: Search strategy for Pubmed (as at 5 October 2017). [file 12981_2019_227_MOESM1_ESM.docx]

| Search | Add to builder | Query | Items found | Time |
| --- | --- | --- | --- | --- |
| [#36](https://www.ncbi.nlm.nih.gov/pubmed/advanced) | [Add](https://www.ncbi.nlm.nih.gov/pubmed/advanced) | Search (((((((((AIDS[Title/Abstract]) OR "Acquired Immunodeficiency Syndrome"[Mesh]) OR AIDS) OR "HIV"[Mesh]) OR Human immunodeficiency virus[Title/Abstract]) OR HIV[Title/Abstract])) AND (((Tenofovir[Title/Abstract]) OR TDF[Title/Abstract]) OR "Tenofovir"[Mesh])) AND (((((((((("Renal Insufficiency"[Mesh]) OR "renal impairment"[Title/Abstract]) OR "kidney impairment"[Title/Abstract]) OR "renal disease"[Title/Abstract]) OR "Nephrotic Syndrome"[Mesh]) OR kidney dysfunction[Title/Abstract]) OR "Kidney Diseases"[Mesh]) OR kidney disease[Title/Abstract]) OR renal insufficiency[Title/Abstract]) OR renal dysfunction[Title/Abstract])) AND (((((Africa[Title/Abstract]) OR "Africa"[Mesh]) OR Africans[Title/Abstract]) OR "African Continental Ancestry Group"[Mesh]) OR "black african"[Title/Abstract]) Filters: Publication date to 2017/10/05 | [53](https://www.ncbi.nlm.nih.gov/pubmed/?cmd=HistorySearch&querykey=36) | 15:03:55 |
| [#3](https://www.ncbi.nlm.nih.gov/pubmed/advanced) | [Add](https://www.ncbi.nlm.nih.gov/pubmed/advanced) | Search (((((((((AIDS[Title/Abstract]) OR "Acquired Immunodeficiency Syndrome"[Mesh]) OR AIDS) OR "HIV"[Mesh]) OR Human immunodeficiency virus[Title/Abstract]) OR HIV[Title/Abstract])) AND (((Tenofovir[Title/Abstract]) OR TDF[Title/Abstract]) OR "Tenofovir"[Mesh])) AND (((((((((("Renal Insufficiency"[Mesh]) OR "renal impairment"[Title/Abstract]) OR "kidney impairment"[Title/Abstract]) OR "renal disease"[Title/Abstract]) OR "Nephrotic Syndrome"[Mesh]) OR kidney dysfunction[Title/Abstract]) OR "Kidney Diseases"[Mesh]) OR kidney disease[Title/Abstract]) OR renal insufficiency[Title/Abstract]) OR renal dysfunction[Title/Abstract])) AND (((((Africa[Title/Abstract]) OR "Africa"[Mesh]) OR Africans[Title/Abstract]) OR "African Continental Ancestry Group"[Mesh]) OR "black african"[Title/Abstract]) | [61](https://www.ncbi.nlm.nih.gov/pubmed/?cmd=HistorySearch&querykey=3) | 15:02:06 |
| [#34](https://www.ncbi.nlm.nih.gov/pubmed/advanced) | [Add](https://www.ncbi.nlm.nih.gov/pubmed/advanced) | Search (((((AIDS[Title/Abstract]) OR "Acquired Immunodeficiency Syndrome"[Mesh]) OR AIDS) OR "HIV"[Mesh]) OR Human immunodeficiency virus[Title/Abstract]) OR HIV[Title/Abstract] | [439669](https://www.ncbi.nlm.nih.gov/pubmed/?cmd=HistorySearch&querykey=34) | 14:53:34 |
| [#33](https://www.ncbi.nlm.nih.gov/pubmed/advanced) | [Add](https://www.ncbi.nlm.nih.gov/pubmed/advanced) | Search AIDS[Title/Abstract] | [143961](https://www.ncbi.nlm.nih.gov/pubmed/?cmd=HistorySearch&querykey=33) | 14:53:17 |
| [#2](https://www.ncbi.nlm.nih.gov/pubmed/advanced) | [Add](https://www.ncbi.nlm.nih.gov/pubmed/advanced) | Search "Acquired Immunodeficiency Syndrome"[Mesh] | [75600](https://www.ncbi.nlm.nih.gov/pubmed/?cmd=HistorySearch&querykey=2) | 14:52:46 |
| [#32](https://www.ncbi.nlm.nih.gov/pubmed/advanced) | [Add](https://www.ncbi.nlm.nih.gov/pubmed/advanced) | Search AIDS | [269901](https://www.ncbi.nlm.nih.gov/pubmed/?cmd=HistorySearch&querykey=32) | 14:52:23 |
| [#31](https://www.ncbi.nlm.nih.gov/pubmed/advanced) | [Add](https://www.ncbi.nlm.nih.gov/pubmed/advanced) | Search "HIV"[Mesh] | [94499](https://www.ncbi.nlm.nih.gov/pubmed/?cmd=HistorySearch&querykey=31) | 14:52:04 |
| [#30](https://www.ncbi.nlm.nih.gov/pubmed/advanced) | [Add](https://www.ncbi.nlm.nih.gov/pubmed/advanced) | Search Human immunodeficiency virus[Title/Abstract] | [84043](https://www.ncbi.nlm.nih.gov/pubmed/?cmd=HistorySearch&querykey=30) | 14:51:41 |
| [#29](https://www.ncbi.nlm.nih.gov/pubmed/advanced) | [Add](https://www.ncbi.nlm.nih.gov/pubmed/advanced) | Search HIV[Title/Abstract] | [296310](https://www.ncbi.nlm.nih.gov/pubmed/?cmd=HistorySearch&querykey=29) | 14:51:23 |
| [#28](https://www.ncbi.nlm.nih.gov/pubmed/advanced) | [Add](https://www.ncbi.nlm.nih.gov/pubmed/advanced) | Search ((Tenofovir[Title/Abstract]) OR TDF[Title/Abstract]) OR "Tenofovir"[Mesh] | [7756](https://www.ncbi.nlm.nih.gov/pubmed/?cmd=HistorySearch&querykey=28) | 14:51:03 |
| [#27](https://www.ncbi.nlm.nih.gov/pubmed/advanced) | [Add](https://www.ncbi.nlm.nih.gov/pubmed/advanced) | Search "Tenofovir"[Mesh] | [3645](https://www.ncbi.nlm.nih.gov/pubmed/?cmd=HistorySearch&querykey=27) | 14:50:35 |
| [#26](https://www.ncbi.nlm.nih.gov/pubmed/advanced) | [Add](https://www.ncbi.nlm.nih.gov/pubmed/advanced) | Search TDF[Title/Abstract] | [2824](https://www.ncbi.nlm.nih.gov/pubmed/?cmd=HistorySearch&querykey=26) | 14:49:58 |
| [#25](https://www.ncbi.nlm.nih.gov/pubmed/advanced) | [Add](https://www.ncbi.nlm.nih.gov/pubmed/advanced) | Search Tenofovir[Title/Abstract] | [5897](https://www.ncbi.nlm.nih.gov/pubmed/?cmd=HistorySearch&querykey=25) | 14:49:36 |
